# Supplementary material for: Improved metagenomic analysis with Kraken 2
Source: Genome Biol. 2019 Nov 28;20:257. doi: 10.1186/s13059-019-1891-0 (PMC6883579; doi:10.1186/s13059-019-1891-0)
Supplement: Supplementary file 2 — Additional file 2: Figure. S1. Estimation of database sizes for Kraken 1 and Kraken 2 as sequences are added to the reference set. Figure S2. Bracken performance on strain exclusion simulated prokaryotic data. Figure S3. Examples of compact hash table usage with Kraken 2. Figure S4. Evaluation of compact hash table error rates as a function of two variables. Figure S5. Evaluation of minimizer collision rates as a function of minimizer length. [file 13059_2019_1891_MOESM2_ESM.docx]

**Supplementary Figures**

for

“Improved metagenomic analysis with Kraken 2”

Derrick E. Wood, Jennifer Lu, and Ben Langmead

**Table of Contents**

Fig. S1. Estimation of database sizes for Kraken 1 and Kraken 2 as sequences are added to the reference set.
Fig. S2. Bracken performance on strain exclusion simulated prokaryotic data.
Fig. S3. Examples of compact hash table usage with Kraken 2.
Fig. S4. Evaluation of compact hash table error rates as a function of two variables.
Fig. S5. Evaluation of minimizer collision rates as a function of minimizer length.


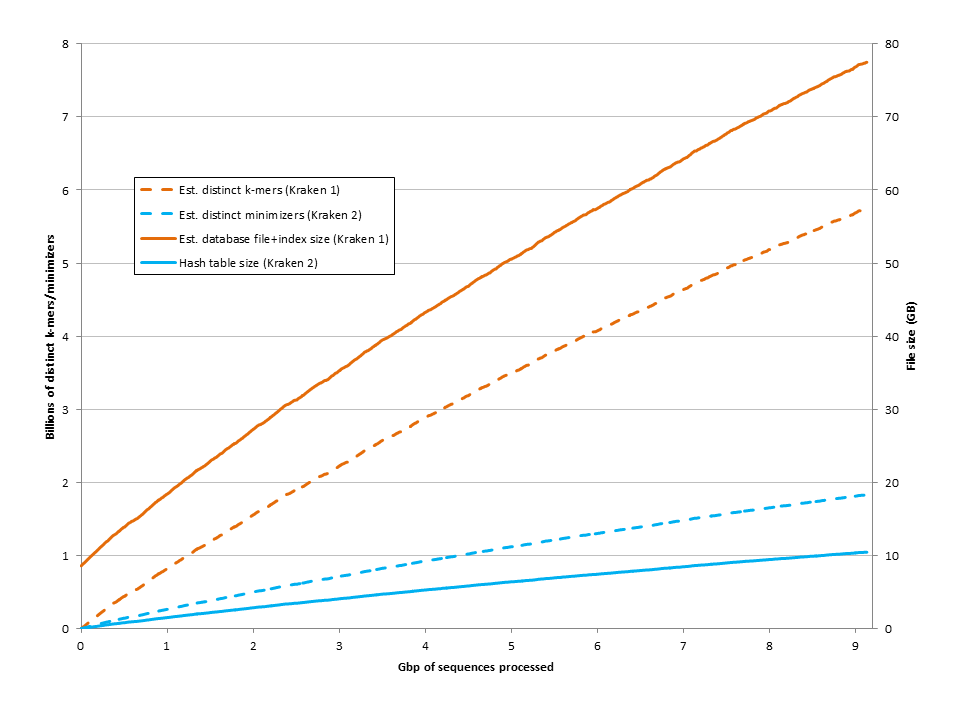


**Fig. S1. Estimation of database sizes for Kraken 1 and Kraken 2 as sequences are added to the reference set.** Over a shuffled set of the nucleotide sequences in our strain exclusion reference, we calculated progressively larger estimates of the number of distinct *k*-mers and minimizers. Because database sizes for Kraken 1 and Kraken 2 are functions of the numbers of distinct *k*-mers and minimizers, respectively, we also calculated the estimated database sizes for Kraken 1 and Kraken 2.


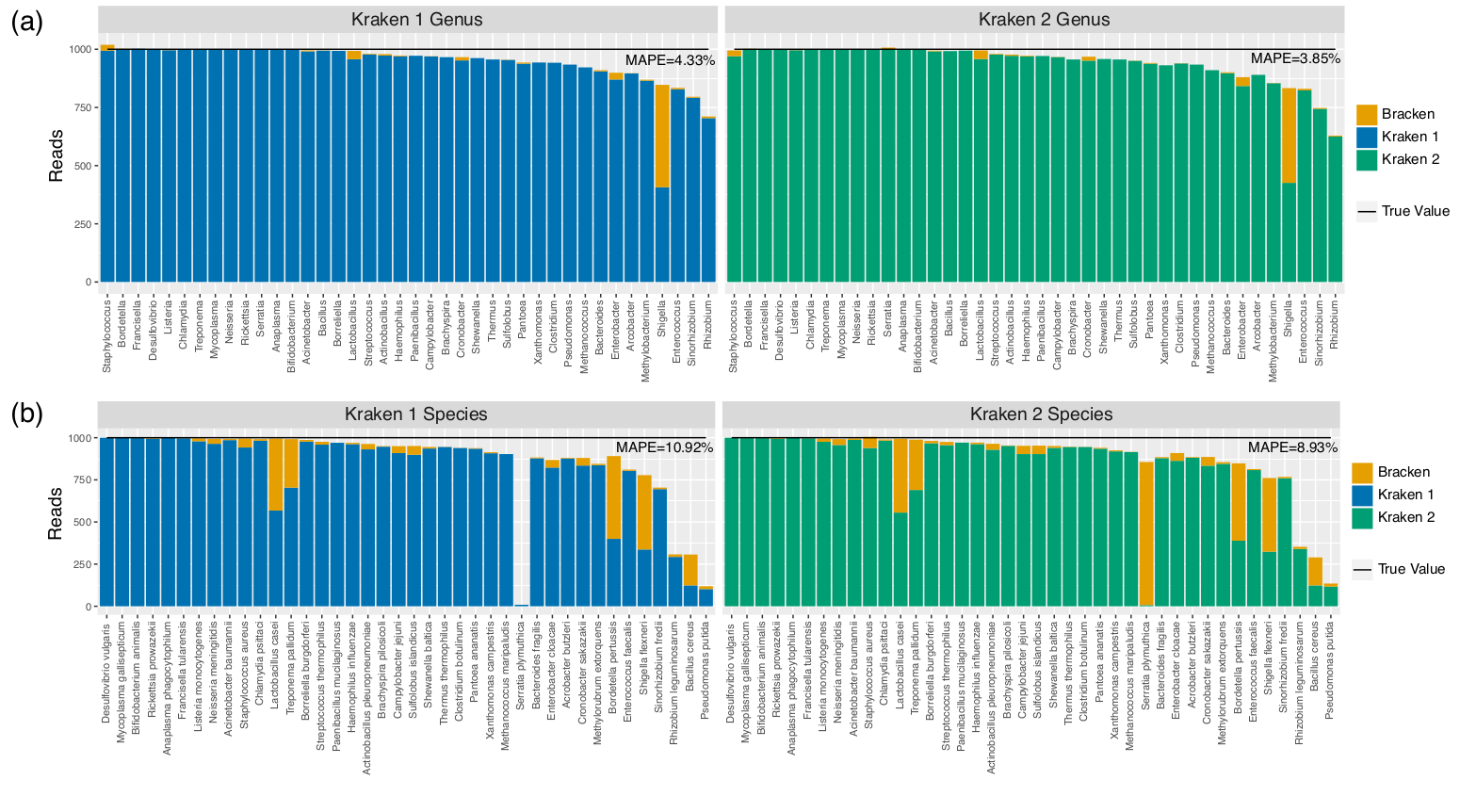


**Fig. S2. Bracken performance on strain exclusion simulated prokaryotic data**. For each of the 40 genomes examined, we removed the genome from the reference genome set, and simulated 1000 paired-end reads from the genome. We used Kraken 1 and Kraken 2 to build databases with the same reference set, and to classify each simulated fragment. We then used Bracken with both programs to estimate **(a)** genus sequence abundance estimation and **(b)** species sequence abundance estimation. “MAPE” is mean absolute percentage error, as defined in the **Methods**.


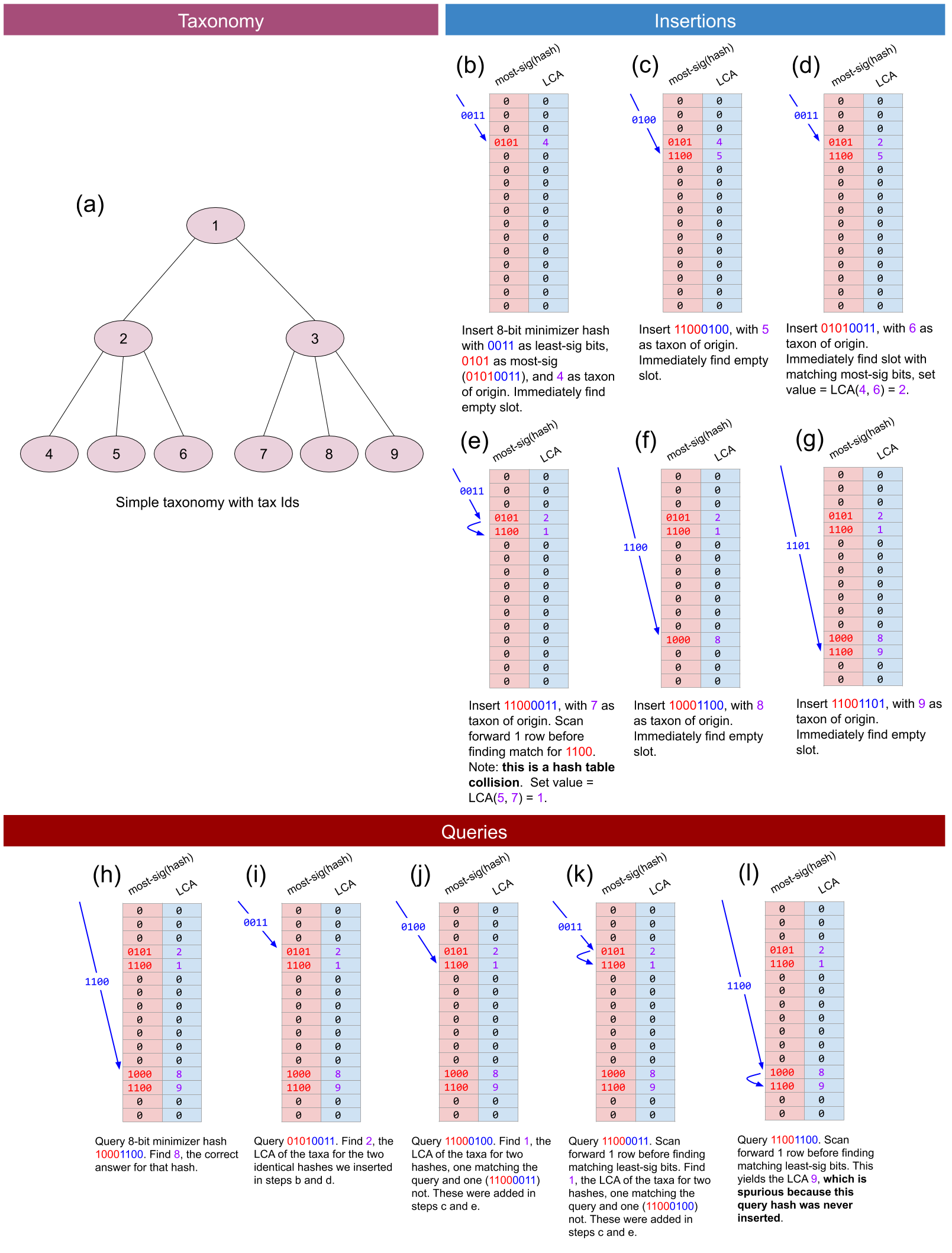


**Fig. S3. Examples of compact hash table usage with Kraken 2.** **(a)** An example of Kraken 2’s reduced internal representation of the taxonomy with sequential ID numbering via breadth-first search. **(b)-(g)** Sequential examples of Kraken 2’s insertion of minimizer/LCA pairs into a compact hash table. **(h)-(l)** Sequential examples of Kraken 2’s querying of a compact hash table with a minimizer. In practice, the table and the lengths of the keys are greater than shown here. Also, since the actual table does not generally have a number of rows equal to a power of 2, we use the modulo operation to obtain the search offset (blue) rather than simply taking bits from the hash value.


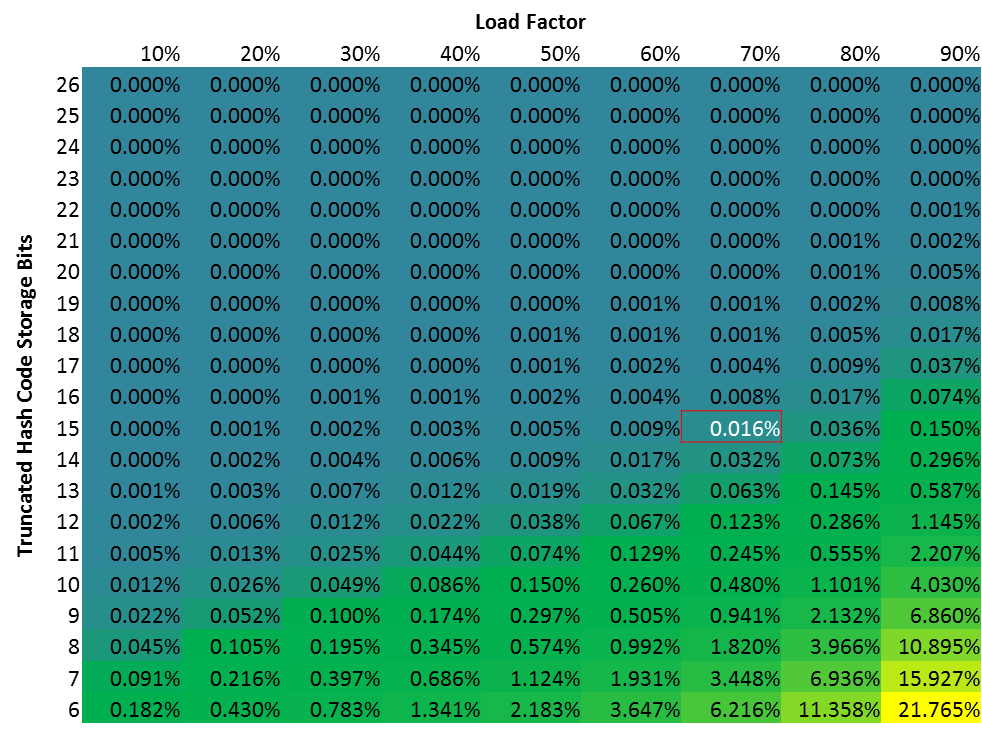


**Fig. S4. Evaluation of compact hash table error rates as a function of two variables.** The error rates of Kraken 2’s compact hash table are a function of the load factor and the number of bits used to store the truncated hash code. Error rates were determined by inserting the minimizers from *P. aeruginosa* UCBPP PA14 and querying with minimizers from randomly generated sequence. Kraken 2’s default database settings used 15 bits to store the truncated hash code and a load factor of 70%, which is highlighted with a red border in the figure.


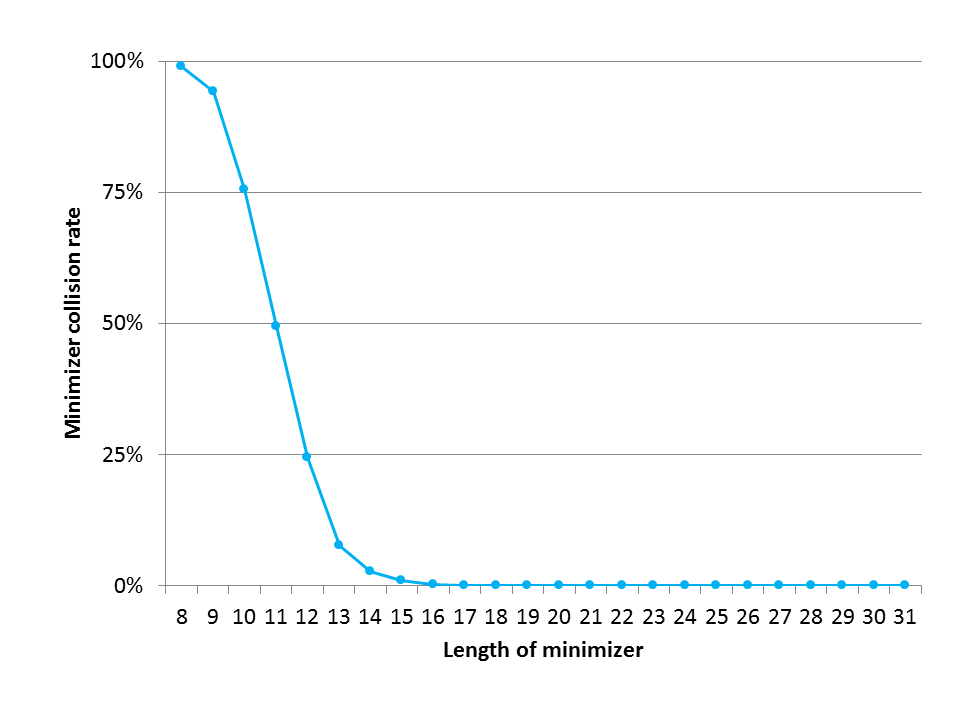


**Fig. S5. Evaluation of minimizer collision rates as a function of minimizer length.** We examined the rate at which minimizers of 35-mers from *P. aeruginosa* UCBPP PA14 would be found as minimizers of 35-mers in randomly generated sequence. Minimizer lengths ℓ were varied from 8 to 31, with no spaced minimizers used. This demonstrates the significantly lower collision rates that occur by use of the long (ℓ=31) minimizers in Kraken 2 versus use of shorter (ℓ <16) minimizers.
