# Supplementary material for: Improved metagenomic analysis with Kraken 2
Source: Genome Biol. 2019 Nov 28;20:257. doi: 10.1186/s13059-019-1891-0 (PMC6883579; doi:10.1186/s13059-019-1891-0)
Supplement: Supplementary file 3 — Additional file 3: Review history. [file 13059_2019_1891_MOESM3_ESM.docx]

Review History

**First round of review**

**Reviewer 1**

**Were you able to assess all statistics in the manuscript, including the appropriateness of statistical tests used?**

No, I do not feel adequately qualified to assess the statistics.

**Comments to author:**

The authors, Wood, Lu, and Langmead, have a well-written and concise manuscript that outlines the improvements made on Kraken 1 to address a shortcoming with the original software, speed and performance. The authors came up with a new approach in Kraken2 for compacting the Kraken library that improves performance without jeopardizing accuracy. The authors go on to compare the speed and accuracy of Kraken 2, Kraken 1, and other time-efficient classification tools. Overall, the manuscript makes an important contribution of a new tool and algorithm to the field of metagenomics classifiers.

Comments for the authors:

Rationale for other pipelines chosen for comparison: there are other classifiers, like MetaPhlAn or MEGAN, that have performed well in speed and accuracy tests, can the authors list their rationale for choosing CLARK and Centrifuge as non-Kraken based pipelines for comparison.

Strain Exclusion experiment:

-	Can the authors include how much additional time running Bracken adds to the overall analysis either in the figure legend or as a separate comparator (Kraken2+Braken) in the Figure 1 tools comparison.

-	Generation of the strain exclusion simulated prokaryotic dataset. The authors should mention more details about how the data set was generated in the methods, particularly that only the chromosomal contig was retained for the generation of artificial reads (or at least that is how I interpreted the comments on the Strain exclusion data generation code on github). Also were the plasmids still included in the reference data? For a genus/species like Shigella flexneri, the only true distinguishing feature from an E. coli is the plasmid it carries. This may lead to some of the poor classification statistics the authors saw for that genus/species. Also it is unclear to the reader why for certain species, like Pseudomonas putida, which should still have at least 2 references in the library, only 1/10th of the reads were classified to the reference library and the authors should address this in the text of their results or discussion.

**Reviewer 2**

**Were you able to assess all statistics in the manuscript, including the appropriateness of statistical tests used?**

Yes, and I have assessed the statistics in my report.

**Comments to author:**

In this manuscript, "Improved metagenomic analysis with Kraken 2", Wood, Lu, and Langmead describe the algorithms, optimizations, classification performance, and computational performance of the new software tool Kraken 2. In all these respects, this represents a significant advancement over the Kraken (v1) tool they released in 2014 (doi:10.1186/gb-2014-15-3-r46) while maintaining the same, underlying search strategy. It is also a significant advance over previously published similar tools (direct comparisons are provided within this manuscript for algorithmically similar tools).

In the past five years, Kraken (v1) has become the de facto standard tool in the field for k-mer based metagenomic classification. However, much of what had reduced its accessibility and usefulness to a wider audience were the extreme memory requirements for its data structures and the inherent sensitivity tradeoffs with the methods available to reduce that footprint. As such, it is highly welcome to see a full rework of the internal memory structures and representation that moves this tool for a typically used database into a much more accessible hardware requirement for the average user.

This manuscript presents a thorough evaluation of Kraken 2's computational performance (Fig 1a), computational resource footprint (Methods), and classification performance, including a comparison of classification performance to the other commonly used k-mer based classifiers (Fig 1b, 1c). I find the results convincing, and am not concerned about the analyses presented in this paper or of the performance of this tool on a technical level. I have been able to download, install, and execute the tool itself and replicate a small portion of the steps taken here. I believe this work to be ready for publication without any significant alterations to the tool or analyses.

However, I do not believe the manuscript itself is quite ready for general consumption and have a number of recommendations on the way the tool--particularly its new behaviors and features as compared to the previous version--is described in the text and figures. Kraken (v1) has built a wide user base over the years, most of whom lack a background in algorithms and data structures, and while much effort is put into describing it in a more understandable manner, it could use more simple anchoring reference points to compare Kraken 2's behavior to what people already know (Kraken 1) on a black box level.

My biggest question is whether the use of the named variables "k-mer" (k) and "minimizer" (l) in this text is potentially misleading or confusing to those who have familiarity with other k-mer based classifiers, such as Kraken 1. In Kraken 1, the value of "k" that a user chooses directly impacts the size of the search key in the database and the sensitivity of the resulting searches. These are k-mers in the traditional search sense. In Kraken 1, the "minimizer", if I understand it correctly, has no impact on the end-to-end outputs of a run--it only acts as an optimization for space and speed, but is irrelevant to classification sensitivity. In Kraken 2, if I understand it correctly, the value of "k" is probably best described as the size of the sliding window in which the actual k-mers are chosen, and the value of "l" is the actual length of the k-mer: the primary variable that affects classification performance. Kraken 2's "k" value is probably best thought of as a space optimization parameter that determines how many adjacent k-mers (of length l) we're allowed to throw away. This discrepancy is most clear in Figure S1, in which the authors compare Kraken 1 and Kraken 2 by plotting v1's "k-mers" against v2's "minimizers". From the point of view of an end-user, it's probably best to make it extremely clear that the "l" knob in v2 is the "k" knob they used to turn in v1 (which would be most clear if v2 renamed "minimizers" to "k-mers" and renamed "k-mers" to "sliding window size").

The spaced seed feature is another new feature in v2 that makes sense to incorporate based on recent work in this area. However, again, since this is a new feature, it is worth providing the comparison points to the v1 tool. It is worth explicitly stating in the methods description portion of the manuscript that Kraken1-like classification behavior is most closely approximated by running Kraken 2 with parameters k=31, l=31, s=0 (this statement is currently buried in the Results in an analysis of database sizes, but not in the Methods section). And that whereas v1's sensitivity profile was mostly a function of k (it's easy for a biologist to conceptually understand that Kraken 1's default k=31 results in sensitivity to approximately 1/k or 3% nucleotide-level genetic distance from the reference database), users should know that v2's sensitivity is a function of l-s (defaulting to 24, or about 4% distance from DB). This intuition is not currently presented in the manuscript.

The section on database sizes, as currently written, states that "the size of a Kraken 2 hash table is a function of the estimate of the number of distinct minimizers in the reference data." This statement though, as well as this section as a whole, doesn't convey an important insight to the user that much of the database size reduction actually comes from the difference between k and l. Again, since the "k-mer" term from Kraken 1 is almost equivalent to the "minimizer" term from Kraken 2, the two leading sentences in this section make it sound like both tools database sizes are primarily a function of the same thing. The difference is that, in v2, we throw away most of the hash keys. The online manual (https://github.com/DerrickWood/kraken2/blob/master/docs/MANUAL.markdown) states it more clearly: "users with low-memory computing environments can replicate the MiniKraken functionality of Kraken 1 in two ways: first, by increasing the value of $k$ with respect to $\ell$". This is the only place in which the importance of the difference of these two values is communicated. There is an existing statement that refers to an empirical 3.1x reduction "for the settings we have selected", but it isn't immediately obvious to a non-technical reader how that changes when they turn any of the three main knobs presented to them.

Currently, the only graphical depiction of the underlying methodology in this manuscript is buried in Figure S2--all other figures are analytic results. Readers could greatly benefit from a simple methodological figure, such as the one presented in the author's tweet (https://twitter.com/DerrickEWood/status/1171427342998278144). This could be a very useful Figure 1.

With the analyses presented, I think the authors do a good job addressing what would otherwise be my primary technical concern about the new data structure: the increased negative impact of hash table collisions in v2 on classification accuracy. While it might be nice to further analyze these impacts in the future sense, given the ever increasing size of available data (in particular, addressing issues in Nasko 2018, doi:10.1186/s13059-018-1554-6), I don't think it's necessary for the scope of this paper and presents opportunities for future work by these or other authors. As long as this manuscript can be improved to clearly explain the conceptual changes from v1 and black box parameter tradeoffs that a computational biologist user would need to know, it should be published quickly without additional analytic work.

**Authors Response**

**Point-by-point responses to the reviewers’ comments:**

*We thank the reviewers for their thoughtful and helpful comments regarding our manuscript. To aid in evaluating our revisions, we provide this summary of changes.*

*Main text:*

*1.	We added a new methodological figure (Figure 1) illustrating Kraken 2’s k-mer classification approach and the impact on speed, memory usage, and accuracy of changing parameters. To comply with the limit on display elements, we moved the previous Figure 2 (Bracken results) to a supplementary figure, and we moved the previous Figure 1 (Tool performance comparison) to Figure 2.*

*2.	We now state explicitly that the genomic substring compared in Kraken 2 is the minimizer, a change from Kraken 1’s use of the k-mer.*

*3.	We now discuss Kraken 2’s classifications of sequences from species with high sequence identity to other species (e.g., those in the Shigella, Bacillus, and Pseudomonas genera).*

*4.	We added a statement of Bracken’s speed.*

*5.	In the tool performance comparison (now Figure 2), we have clarified the legend to show that it is a comparison of sequence classification tools, and that we evaluated accuracy on a per-fragment basis.*

*Methods:*

*6.	Within the “Minimizer-based subsampling” section, we now note that as k increases with respect to ℓ, database size, memory usage, and runtime all decrease.*

*7.	Within the “Spaced seed usage” section, we now explicitly discuss the similarity between Kraken 1’s operation and Kraken 2’s with k=ℓ=31 & s=0.*

*8.	Also within the “Spaced seed usage” section, we now discuss the impact on sensitivity of the ℓ and s parameters.*

*9.	Within the “Classification of a sequence fragment with Kraken 2” section, we added clarifying text as to the role of k-mers within Kraken 2.*

*10.	Within the “Generation of data for strain exclusion experiments” section, we added clarifying text as to the selection of genomes and construction of reference/simulated read data.*

*11.	Within the “Execution of strain exclusion experiments” section, we added discussion on our criteria for selecting comparator programs, as well as discussion on the suitability of comparing against abundance estimation programs.*

*12.	We made other changes regarding data availability statements, persistent data identifiers, and other required sections to comply with journal guidelines.*

*Additionally, minor changes to wording or figures were made, such as the use of “F1-measure” instead of “F-measure” and “open-source” instead of “open-access”. All text differences should be highlighted in the text; no changes have been made to our results. Tables of contents have also been added to our supplementary material. Our point-by-point responses to reviewers follow.*

Reviewer #1: The authors, Wood, Lu, and Langmead, have a well-written and concise manuscript that outlines the improvements made on Kraken 1 to address a shortcoming with the original software, speed and performance. The authors came up with a new approach in Kraken2 for compacting the Kraken library that improves performance without jeopardizing accuracy. The authors go on to compare the speed and accuracy of Kraken 2, Kraken 1, and other time-efficient classification tools. Overall, the manuscript makes an important contribution of a new tool and algorithm to the field of metagenomics classifiers.

Comments for the authors:

Rationale for other pipelines chosen for comparison: there are other classifiers, like MetaPhlAn or MEGAN, that have performed well in speed and accuracy tests, can the authors list their rationale for choosing CLARK and Centrifuge as non-Kraken based pipelines for comparison.

Authors’ Response: We added our selection criteria to the manuscript (Change #11 in our summary). As noted, we compared against other fast classification tools, i.e., roughly within an order of magnitude of Kraken’s speed. Further, we only considered tools that (a) perform classification of shotgun-sequenced fragments from entire genomes (rather than just marker genes), and (b) support customization of the reference database. Two programs in the reviewer’s list do not meet these criteria. MetaPhlAn is a population abundance estimation program that would perform poorly in the kind of comparison we perform here due to a low proportion of classified fragments (see Table 1 of Lindgreen, et al. doi: 10.1038/srep19233). Additionally, we have already compared Kraken 1’s speed directly against MetaPhlAn’s in the first Kraken paper, and discussed the differences between abundance estimation programs and Kraken in that paper as well. Lindgreen, et al. also show that MEGAN was approximately 40 times slower than Kraken 1, putting it well outside the “order of magnitude” guideline we set. MEGAN was run with the DIAMOND aligner in that comparison, and we note that in the original Kraken paper we found Kraken 1 to be 210-315 times faster than MEGABLAST (the fastest of the BLAST tools) so any use of a BLAST-based alignment tool with MEGAN would also be substantially slower than Kraken 1.

Strain Exclusion experiment:

- Can the authors include how much additional time running Bracken adds to the overall analysis either in the figure legend or as a separate comparator (Kraken2+Braken) in the Figure 1 tools comparison.

*Authors’ Response: We added a statement about Bracken’s runtime (which is less than one second) to the main text (Change #4). Since Bracken’s function is to produce sequence abundance estimates – not taxonomic assignments for reads (Change #11) -- we decided not to include it in the tool performance comparison figure (now Figure 2). We modified the figure legend to clarify that the comparison is of sequence classification tools (Change #5).*

- Generation of the strain exclusion simulated prokaryotic dataset. The authors should mention more details about how the data set was generated in the methods, particularly that only the chromosomal contig was retained for the generation of artificial reads (or at least that is how I interpreted the comments on the Strain exclusion data generation code on github). Also were the plasmids still included in the reference data? For a genus/species like Shigella flexneri, the only true distinguishing feature from an E. coli is the plasmid it carries. This may lead to some of the poor classification statistics the authors saw for that genus/species.

*Authors’ Response: We agree that this was confusing, and have taken steps to clarify the matter. We added text to the manuscript to provide more details regarding dataset generation (see Change #10) and added comments to the data generation code itself. In our experiments, we simulate reads from plasmids only in cases where those plasmids were associated with the strain of origin in the taxonomy we obtained from NCBI. In the case of S. flexneri, for example, the plasmids are indeed used to simulate reads. Additionally, if plasmids are not associated with one of our excluded strains, then they would be included in the strain exclusion reference data. Thus, the inclusion or exclusion of a sequence depends on the NCBI-provided taxonomy assignments, but all downloaded genomic sequences were either in the reference set or were the basis for simulated read data.*

*During our re-examination of the code, we fixed a README file that had contained some incorrect file names. We thank the reviewer for asking for this enhanced discussion on the methods used to generate the data, and so bringing these errors to our attention.*

*Regarding classification statistics for Shigella, we note that fragments from genomic regions that are identical between members of two genera will not be able to be classified at a species or genus level of the taxonomy. In Kraken, such fragments will likely be classified at a family level, the lowest level possible given sequence identity across genera. As the reviewer notes, Shigella and E. coli could (according to sequence identity) be considered a single species, and if such a taxonomy change were to occur, the Kraken (and other classifiers’) genus-level sensitivity would be considerably higher for those species. Related discussion has been added to the manuscript around the effects of high identity between species on classification (see Change #3 and the reviewer’s next point).*

Also it is unclear to the reader why for certain species, like Pseudomonas putida, which should still have at least 2 references in the library, only 1/10th of the reads were classified to the reference library and the authors should address this in the text of their results or discussion.

*Authors’ Response: We agree that this point needed further clarification. We added discussion of this (see Change #3). Further, we re-examined the underlying Kraken 2 classification output, and found nearly 70% of the simulated P. putida reads were actually classified to a sister species, P. monteilii. The 10% that the reviewer refers to reflects only those reads that were classified directly to the P. putida species. As we now cite in our revised text, Pseudomonas is known to have high sequence identity between species and strains across species, as a result of taxonomic inconsistency (Gomila, et al. doi: 10.3389/fmicb.2015.00214), which can affect classification results negatively.*

Reviewer #2: In this manuscript, "Improved metagenomic analysis with Kraken 2", Wood, Lu, and Langmead describe the algorithms, optimizations, classification performance, and computational performance of the new software tool Kraken 2. In all these respects, this represents a significant advancement over the Kraken (v1) tool they released in 2014 (doi:10.1186/gb-2014-15-3-r46) while maintaining the same, underlying search strategy. It is also a significant advance over previously published similar tools (direct comparisons are provided within this manuscript for algorithmically similar tools).

In the past five years, Kraken (v1) has become the de facto standard tool in the field for k-mer based metagenomic classification. However, much of what had reduced its accessibility and usefulness to a wider audience were the extreme memory requirements for its data structures and the inherent sensitivity tradeoffs with the methods available to reduce that footprint. As such, it is highly welcome to see a full rework of the internal memory structures and representation that moves this tool for a typically used database into a much more accessible hardware requirement for the average user.

This manuscript presents a thorough evaluation of Kraken 2's computational performance (Fig 1a), computational resource footprint (Methods), and classification performance, including a comparison of classification performance to the other commonly used k-mer based classifiers (Fig 1b, 1c). I find the results convincing, and am not concerned about the analyses presented in this paper or of the performance of this tool on a technical level. I have been able to download, install, and execute the tool itself and replicate a small portion of the steps taken here. I believe this work to be ready for publication without any significant alterations to the tool or analyses.

However, I do not believe the manuscript itself is quite ready for general consumption and have a number of recommendations on the way the tool--particularly its new behaviors and features as compared to the previous version--is described in the text and figures. Kraken (v1) has built a wide user base over the years, most of whom lack a background in algorithms and data structures, and while much effort is put into describing it in a more understandable manner, it could use more simple anchoring reference points to compare Kraken 2's behavior to what people already know (Kraken 1) on a black box level.

My biggest question is whether the use of the named variables "k-mer" (k) and "minimizer" (l) in this text is potentially misleading or confusing to those who have familiarity with other k-mer based classifiers, such as Kraken 1. In Kraken 1, the value of "k" that a user chooses directly impacts the size of the search key in the database and the sensitivity of the resulting searches. These are k-mers in the traditional search sense. In Kraken 1, the "minimizer", if I understand it correctly, has no impact on the end-to-end outputs of a run--it only acts as an optimization for space and speed, but is irrelevant to classification sensitivity. In Kraken 2, if I understand it correctly, the value of "k" is probably best described as the size of the sliding window in which the actual k-mers are chosen, and the value of "l" is the actual length of the k-mer: the primary variable that affects classification performance. Kraken 2's "k" value is probably best thought of as a space optimization parameter that determines how many adjacent k-mers (of length l) we're allowed to throw away. This discrepancy is most clear in Figure S1, in which the authors compare Kraken 1 and Kraken 2 by plotting v1's "k-mers" against v2's "minimizers". From the point of view of an end-user, it's probably best to make it extremely clear that the "l" knob in v2 is the "k" knob they used to turn in v1 (which would be most clear if v2 renamed "minimizers" to "k-mers" and renamed "k-mers" to "sliding window size").

*Authors’ Response: We agree that it is important to be clear about the connection between Kraken 2’s minimizers and Kraken 1’s k-mers. We clarified this in our revised manuscript through several changes, including the introduction of a new methodological figure (Change #1 in our summary), as well as text in various places in the manuscript (Changes #2 and #9). Because the term “minimizer” also refers to the method by which the compared substrings are selected, and because the k-mer continues to have a role in Kraken 2’s classification algorithm (see Change #9), we did not rename “k-mer” to “sliding window size” as suggested. Nonetheless, we have considerable appreciation for the reviewer’s point, and believe that our changes do much to clarify the connection between the two versions’ parameters.*

The spaced seed feature is another new feature in v2 that makes sense to incorporate based on recent work in this area. However, again, since this is a new feature, it is worth providing the comparison points to the v1 tool. It is worth explicitly stating in the methods description portion of the manuscript that Kraken1-like classification behavior is most closely approximated by running Kraken 2 with parameters k=31, l=31, s=0 (this statement is currently buried in the Results in an analysis of database sizes, but not in the Methods section). And that whereas v1's sensitivity profile was mostly a function of k (it's easy for a biologist to conceptually understand that Kraken 1's default k=31 results in sensitivity to approximately 1/k or 3% nucleotide-level genetic distance from the reference database), users should know that v2's sensitivity is a function of l-s (defaulting to 24, or about 4% distance from DB). This intuition is not currently presented in the manuscript.

*Authors’ Response: We added explicit discussion about these points (see Changes #7 and #8) to the section on spaced seeds. Additionally, panel (e) of our new Figure 1 (see Change #1) shows the relationship between s and both sensitivity and PPV, to better inform users of the parameter tradeoffs.*

The section on database sizes, as currently written, states that "the size of a Kraken 2 hash table is a function of the estimate of the number of distinct minimizers in the reference data." This statement though, as well as this section as a whole, doesn't convey an important insight to the user that much of the database size reduction actually comes from the difference between k and l. Again, since the "k-mer" term from Kraken 1 is almost equivalent to the "minimizer" term from Kraken 2, the two leading sentences in this section make it sound like both tools database sizes are primarily a function of the same thing. The difference is that, in v2, we throw away most of the hash keys. The online manual (https://github.com/DerrickWood/kraken2/blob/master/docs/MANUAL.markdown) states it more clearly: "users with low-memory computing environments can replicate the MiniKraken functionality of Kraken 1 in two ways: first, by increasing the value of $k$ with respect to $\ell$". This is the only place in which the importance of the difference of these two values is communicated. There is an existing statement that refers to an empirical 3.1x reduction "for the settings we have selected", but it isn't immediately obvious to a non-technical reader how that changes when they turn any of the three main knobs presented to them.

*Authors’ Response: We have added explicit discussion about this point (see Change #6) to the section on minimizer-based subsampling. Additionally, panel (d) of our new Figure 1 (Change #1) shows the relationship between (k- ℓ) and each of speed, memory, and accuracy, to better inform users of the parameter tradeoffs.*

Currently, the only graphical depiction of the underlying methodology in this manuscript is buried in Figure S2--all other figures are analytic results. Readers could greatly benefit from a simple methodological figure, such as the one presented in the author's tweet (https://twitter.com/DerrickEWood/status/1171427342998278144). This could be a very useful Figure 1.

*Authors’ Response: We thank the reviewer for this suggestion. We added a modified version of that figure, along with several graphs illustrating the effects of changes to various parameters, as the new Figure 1 (see Change #1).*

With the analyses presented, I think the authors do a good job addressing what would otherwise be my primary technical concern about the new data structure: the increased negative impact of hash table collisions in v2 on classification accuracy. While it might be nice to further analyze these impacts in the future sense, given the ever increasing size of available data (in particular, addressing issues in Nasko 2018, doi:10.1186/s13059-018-1554-6), I don't think it's necessary for the scope of this paper and presents opportunities for future work by these or other authors. As long as this manuscript can be improved to clearly explain the conceptual changes from v1 and black box parameter tradeoffs that a computational biologist user would need to know, it should be published quickly without additional analytic work.

*Authors’ Response: We thank the reviewer for their kind comments.*

**Second round of review**

**Reviewer 1**

The authors addressed all my previous comments. No additional comments on the manuscript.

**Reviewer 2**

I thank the authors for thoroughly revising text and visuals to address the various issues I had raised.

The new Figure 1 goes a very long way in conveying the method to readers, as does the added bits of clarification and reemphasis throughout the text. It helps to illustrate the methods that are otherwise only described in text, with particular attention to the user-adjustable parameters that affect performance. One possible tweak might be to horizontally flip the x-axis of the plots in Figure 1e and label them as $\ell - s$ from 24 to 31 (same data, and the caption would make clear that this plot is for fixed $\ell$, not exploring the entire combinatorial space) -- the point being to convey to the reader that this is the primary value that affects sensitivity and PPV. However, I'm also fine if the authors would prefer to keep it as is.

I appreciate the author's points on the terminology around k-mers and minimizers--and didn't realize some of the subtleties on this point related to the additional text added on page 8--and am happy with how the language here is made explicitly clear and how the comparison points to Kraken 1 (who most readers will be mentally comparing this to) have been clarified.

I believe this is ready to publish quickly.
